# Changes in hypothalamic subunits volume and their association with metabolic parameters and gastrointestinal appetite-regulating hormones following bariatric surgery

**DOI:** 10.1162/IMAG.a.970

**Published:** 2025-10-31

**Authors:** Amélie Lachance, Justine Daoust, Mélissa Pelletier, Alexandre Caron, André C. Carpentier, Laurent Biertho, Josefina Maranzano, André Tchernof, Mahsa Dadar, Andréanne Michaud

**Affiliations:** Centre Nutrition, Santé et Société (NUTRISS), Institut sur la nutrition et les aliments fonctionnels (INAF), Université Laval, Québec, Canada; Institut universitaire de cardiologie et de pneumologie de Québec-Université Laval, Québec, Canada; École de nutrition, Faculté des sciences de l’agriculture et de l’alimentation, Université Laval, Québec, Canada; Centre de recherche du centre hospitalier universitaire de Sherbrooke, Université de Sherbrooke, Sherbrooke, Canada; Département de chirurgie générale, Institut universitaire de cardiologie et de pneumologie de Québec-Université Laval, Québec, Canada; Département d’anatomie, Université du Québec à Trois-Rivières, Trois-Rivières, Canada; Douglas Research Centre, Université McGill, Montréal, Canada

**Keywords:** hypothalamus, structural MRI, volumetric study, segmentation, bariatric surgery, gastrointestinal appetite-regulating hormones

## Abstract

Some nuclei of the hypothalamus are known for their important roles in maintaining energy homeostasis and regulating food intake. Moreover, obesity has been associated with hypothalamic inflammation and morphological alterations, as indicated by increased volume. However, the reversibility of these changes after bariatric surgery-induced weight loss remains underexplored. The aim of this study was to characterize volume changes in hypothalamic subunits up to 2 years following bariatric surgery. A secondary objective was to explore whether changes in hypothalamic subunit volumes were associated with changes in metabolic parameters and levels of gastrointestinal appetite-regulating hormones. Participants with severe obesity undergoing bariatric surgery were recruited. They completed high-resolution T1-weighted brain magnetic resonance imaging (MRI) before bariatric surgery and at 4, 12, and 24 months post-surgery. Blood samples collected during the fasting and postprandial states were analyzed for concentrations of glucagon-like peptide 1 (GLP-1), peptide YY (PYY), and ghrelin. The hypothalamus was segmented into five subunits per hemisphere using a publicly available automated tool. Linear mixed-effects models were employed to examine volume changes between visits and their associations with variables of interest. A total of 73 participants (mean age 44.5 ± 9.1 years; mean body mass index (BMI) 43.5 ± 4.1 kg/m^2^) were included at baseline, with 22 participants completing 24-month follow-up. Significant volume reductions were observed in the whole left hypothalamus 24 months post-surgery. More specifically, decreases were noted in both the left anterior-superior and left posterior subunits at 12 and 24 months post-surgery (all p < 0.05, after false discovery rate (FDR) correction). Smaller volumes in these subunits were significantly associated with a greater percentage of total weight loss (both subunits p < 0.001), as well as with higher postprandial PYY levels (both subunits p < 0.05). These findings suggest that some hypothalamic morphological alterations observed in the context of obesity could potentially be reversed following bariatric surgery-induced weight loss.

## 1 Introduction

Food intake involves a complex, simultaneous interplay of various brain regions (Dagher, 2012). Despite its modest 1% contribution to total brain weight on average, the hypothalamus plays a central role in regulating this process (Dagher, 2012; Saper, 2012). Acting as a control center, it integrates signals from a myriad of afferent nerves within the body and coordinates their transmission to cortical and subcortical grey matter (Saper & Lowell, 2014). Therefore, the hypothalamus contributes to several critical biological mechanisms essential for basic survival functions, including energy metabolism and the homeostatic control of food intake (Saper & Lowell, 2014). These functions are orchestrated by a dozen specific nuclei within the hypothalamus, which are characterized by distinct cellular populations (Bedont et al., 2015; Billot et al., 2020; Xie & Dorsky, 2017). Among these are the arcuate, lateral, paraventricular, dorsomedial, and ventromedial nuclei, all implicated in the regulation of food intake (Caron & Richard, 2017).

Given its fundamental role in metabolic homeostasis, the hypothalamus has been extensively studied to elucidate potential disruptions associated with obesity. Most of these studies have been conducted in animal models, with relatively limited investigations conducted in humans. Challenges associated with the small size of the hypothalamus have limited the use of magnetic resonance imaging (MRI) techniques until recent advancements. Manual delineation of its boundaries, which requires expertise and a valuable amount of time (Schindler et al., 2013), has now been supplanted by the development of automated segmentation techniques, facilitating the study of larger cohorts (Billot et al., 2020; Rodrigues et al., 2022). The automated segmentation tool developed by Billot et al. (2020) divides the hypothalamus into five subunits per hemisphere, offering nuanced insights into its functioning (Billot et al., 2020). Using this tool, two recent studies showed morphological alterations in the hypothalamus among individuals living with overweight or obesity, with increased volume for the whole hypothalamus and for specific subunits (Alzaid et al., 2024; Brown et al., 2023). However, the mechanisms underlying these findings and whether these hypothalamic alterations in individuals with obesity are permanent or reversible following interventions targeting weight loss and cardiometabolic improvements remain unexplored.

Bariatric surgery is known to induce significant and sustained weight loss, along with metabolic improvements, although the magnitude of these effects can vary depending on the type of surgery (Chang et al., 2014; Iqbal et al., 2020). Therefore, it presents a unique opportunity to study long-term effects of substantial weight loss on brain integrity in a prospective setting (Maciejewski et al., 2016; O’Brien et al., 2019; Salminen et al., 2022). Significant changes in both white and grey matter densities have been documented post-bariatric surgery (Michaud et al., 2020; Rullmann et al., 2018; Tuulari et al., 2016; Wang et al., 2020). Previous studies have also examined the impact of bariatric surgery on hypothalamic morphological changes using voxel-based morphometry (VBM), with only one reporting volume changes in specific subregions (Rullmann et al., 2019; van de Sande-Lee et al., 2020). A recent study using an automated segmentation tool, which is considered more reliable than VBM techniques for assessing volume in such a small region of the brain (Arkink et al., 2017), demonstrated reductions in hypothalamic volume 1 year post-surgery, particularly in different subunits of the left hypothalamus (Pané et al., 2024). However, a study with longer-term follow-up is needed to explore the sustainability of the effects of weight loss and concomitant cardiometabolic improvements on hypothalamic morphometric changes.

The mechanisms underlying hypothalamic morphological alterations in the context of obesity remain unclear. Various hypotheses have been explored over the years, including hypothalamic inflammation and gliosis (Schur et al., 2015; Sewaybricker et al., 2023; Thaler et al., 2013) as well as a decreased level of brain vascularization and cerebral hypoperfusion (Alfaro et al., 2018; Bettcher et al., 2013; Willeumier et al., 2011). Some gastrointestinal hormones could also play a role in the remodeling process of the hypothalamus, as they exert their effects by binding to receptors within the hypothalamus (Al Massadi et al., 2017; Farr et al., 2016; Jensen et al., 2018; Karra et al., 2009; Li et al., 2023) or by acting on neurons projecting into hypothalamic nuclei (Biddinger et al., 2020). Moreover, concomitant changes in hunger and satiety hormones have been reported in individuals with obesity, including increased fasting and postprandial ghrelin levels, along with decreased postprandial glucagon-like peptide 1 (GLP-1) and peptide YY (PYY) levels (Aukan et al., 2023). Changes in theses hormones have also been observed following bariatric surgery (Huang et al., 2023). It remains unknown how changes in peripheral gut hormones are associated with hypothalamic morphometric changes following bariatric surgery.

In this prospective study, we aimed to characterize changes in hypothalamic volume and its subunits up to 2 years following bariatric surgery using an automated segmentation tool (Billot et al., 2020). We hypothesized that bariatric surgery leads to reductions in total left and right hypothalamus volume and their specific subunits. As a secondary, exploratory objective, we also examined potential associations between hypothalamic volume changes, regardless of their statistical significance, and the magnitude of weight loss, as well as improvements in cardiometabolic parameters and gastrointestinal appetite-regulating hormone levels. Specifically, we focused on changes in fasting ghrelin and postprandial GLP-1 and PYY, as we hypothesized that these hormones will be more strongly associated with hypothalamic volume when their orexigenic and anorexigenic effects, respectively, are most pronounced.

## 2 Methods

### 2.1 Participants

Ninety-two participants scheduled to undergo bariatric surgery at the *Institut universitaire de cardiologie et de pneumologie de Québec-Université Laval* (IUCPQ-UL) in Québec, Canada, were recruited (Supplementary Fig. S1). These participants were part of a larger prospective study, which aimed at investigating the determinants of metabolic recovery following bariatric surgeries. The protocol has been previously described in detail (Michaud et al., 2020). Briefly, participants had to be 18 to 60 years old and meet the NIH Guidelines for bariatric surgery (“Gastrointestinal surgery for severe obesity: National Institutes of Health Consensus Development Conference Statement,” 1992). Exclusion criteria included gastrointestinal diseases, such as irritable bowel syndrome or gastro-intestinal ulcers, cirrhosis or albumin deficiency, neurological illnesses, uncontrolled high blood pressure, use of any medication that can affect the central nervous system, previous bariatric or brain surgery, severe food allergy, current pregnancy, and substance or alcohol abuse. Specifically, participants were asked to abstain from substance use and to limit alcohol consumption to no more than 2–3 drinks per week to be eligible for bariatric surgery. Participants with any contraindications for MRI, including claustrophobia, metal embedded in the body, or the presence of an implanted medical device, were also excluded.

Furthermore, a subset of participants served as a control group to assess the reliability of the segmentation tool and to ensure that the observed changes in volume were attributable to surgery rather than time or variability in segmentation results. This control group (n = 19) was part of a larger study and underwent two MRI sessions prior to surgery (approximately at -4 and -1 months). The same study design, including the acquisition protocol and the segmentation procedures, was applied. The characteristics of this group have been previously reported (Legault et al., 2024). One participant of the control group had a considerable weight gain between both pre-surgery visits (∆ 5.4 kg, +3.9% of the initial body weight) and was, therefore, excluded from further analyses. The protocol was approved by the research ethics committee of the *Centre de recherche de l’IUCPQ-UL* (no. 2016-2569). A written consent form was signed by each participant at their first visit.

### 2.2 Surgical procedures

Participants received either a sleeve gastrectomy (SG), a Roux-en-Y gastric bypass (RYGB), or a biliopancreatic diversion with duodenal switch (BPD-DS). All surgeries were performed laparoscopically. Regarding SG, a 250 cm^3^ vertical gastrectomy was performed with a 34–44 French Bougie starting 7–8 cm from the pylorus to the His angle (Biertho et al., 2016). For the RYGB, a 30–50 cm^3^ proximal gastric pouch was first created and then connected to the proximal small intestine by bypassing the first 100 cm (Zeighami et al., 2021). Finally, the BPD-DS was performed by first doing a 250 cm^3^ SG and, thereafter, the duodenum was transected about 4 cm distal from the pylorus and anastomosed to a 250-cm alimentary limb, with a 100-cm common channel (Biertho et al., 2010).

### 2.3 Study design and experimental procedures

Outcomes were assessed prior to surgery, and at 4, 12, and 24 months postoperatively. An identical protocol was followed at each visit. Participants were asked to fast for 12 hours overnight before attending the study visit the following morning. Weight and body composition were measured by bioimpedance (InBody520, body composition analyzer, Biospace, Los Angeles, California or Tanita DC-430U, Arlington Heights, IL). Anthropometric measures (waist, hip, and neck circumferences) and blood pressure (systolic (SBP) and diastolic (DBP)) were measured using standardized procedures (Michaud et al., 2020). The percentage of total weight loss (%TWL; [(baseline weight – follow-up weight)/baseline weight] x 100%) and the percentage of excess weight loss (%EWL; [(baseline weight – follow-up weight)/(pre-surgery weight − ideal weight for a body mass index (BMI) of 23 kg/m^2^)] x 100%) were calculated. Medical history, current health status, medication use, surgical complications, and substance and alcohol consumption data were recorded by a trained research assistant.

### 2.4 Plasma lipid profile, glucose homeostasis, and gastrointestinal appetite-regulating hormones

Blood samples were taken after a 12-hour overnight fast and approximately 1 hour after the consumption of a standard nutritional beverage (237 ml Boost Original, Nestlé Health Science (240 kcal, 41 g of carbohydrates, 10 g of proteins, and 4 g of lipids)). Blood samples were collected into chilled tubes with appropriate enzymatic inhibitors (dipeptidyl peptidase-IV inhibitor, aprotinin, or HCl) (Millipore Sigma Canada, Ontario). All samples were immediately kept at 4ºC before being centrifuged and then stored at -80ºC. Fasted acylated and non-acylated ghrelin were measured using ELISA kits (Bertin Pharma; A05106 and A05119, respectively) and summed to obtain total ghrelin. Levels of post-prandial GLP-1 and postprandial PYY were measured by multiplex assay (Millipore Sigma Canada, Ontario; HMH3-34K) (Richard et al., 2022). Plasma levels of cholesterol, high-density lipoproteins (HDL), low-density lipoproteins (LDL), triglycerides, glucose, and insulin were measured at each visit as part of the routine monitoring of patients. The homeostatic model assessment for insulin resistance (HOMA-IR) was calculated (fasting glucose (mmol/L) x fasting insulin (pmol/L) / 135) (Matthews et al., 1985).

### 2.5 MRI acquisition

Participants underwent an MRI session approximately 1 to 2 hours after consuming the standardized nutritional beverage to control for food and fluid intake across individuals. T1-weighted three-dimensional (3D) turbo echo images of the brain were acquired using a 3T whole-body MRI scanner (Philips, Ingenia, Philips Medical Systems) equipped with a 32-channel head coil at the *Centre de recherche de l’IUCPQ-UL*. The following parameters were used: 176 sagittal 1.0 mm slices, TR/TE = 8.1/3.7 ms, field of view (FOV) = 240 x 240 mm^2^, and voxel size = 1 x 1 x 1 mm.

### 2.6 MRI data analysis and hypothalamus segmentation

**Fig. 1.**
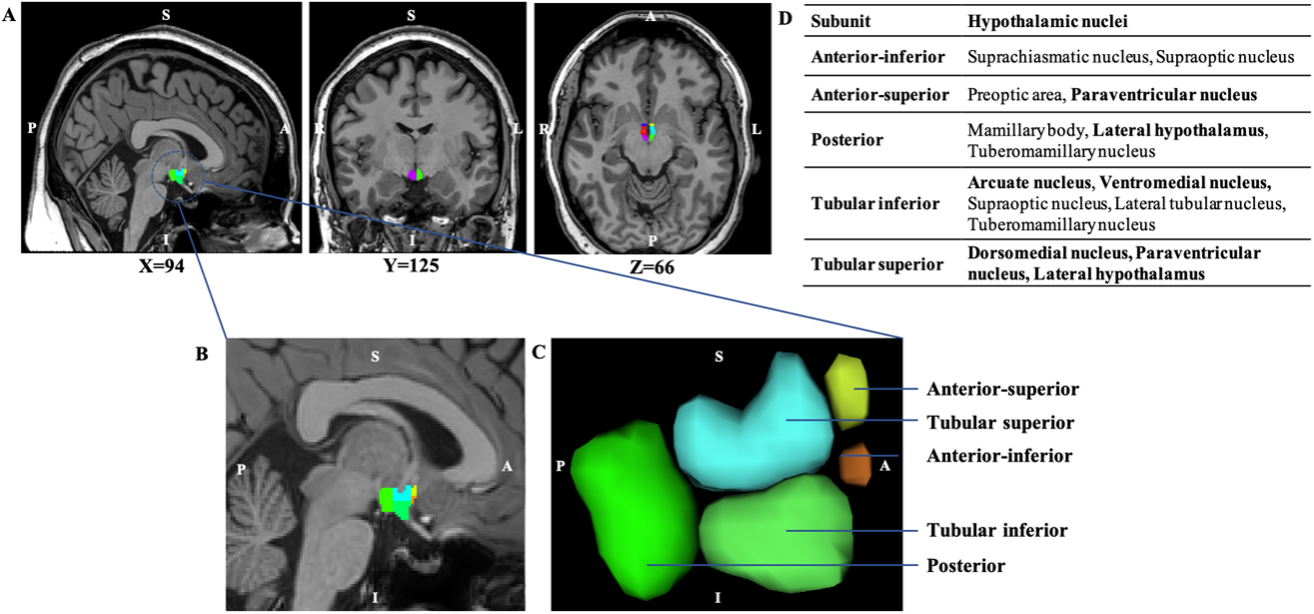
Visual representation of the hypothalamic segmentation. (A) Sagittal, coronal, and axial brain slices showing hypothalamic segmentation and different hypothalamic subunits. (B) Expanded view of the sagittal plane showing the left hypothalamus. (C) Three-dimensional view of the left hypothalamus. (D) Tables presenting the five hypothalamic subunits, and the corresponding hypothalamic nuclei included within them. Principal nuclei involved in food intake are highlighted in bold. “L” denotes left and “R” denotes right. “P” denotes posterior, “A” denotes anterior, “S” denotes superior, and “I” denotes inferior.

Standard preprocessing steps, including image denoising (Coupe et al., 2008), intensity non-uniformity correction (Sled, Zijdenbos, & Evans, 1998), and image intensity normalization into range (0–100) using histogram matching, were first applied. Images were then linearly (using a nine-parameter rigid registration) registered to an average brain template (MNI ICBM152-2009c) using MNI MINC tools (https://www.bic.mni.mcgill.ca/ServicesSoftware/MINC). Hypothalamus segmentation was performed on the standardized images using an open-source, automated method based on a deep convolutional neural network (Billot et al., 2020), available as part of the FreeSurfer brain segmentation software. This segmentation tool has been previously validated and used to perform hypothalamus segmentation in similar applications (Alzaid et al., 2024; Brown et al., 2023; Pané et al., 2024; Ruzok et al., 2022). This tool allows the segmentation of the hypothalamus in five subunits per hemisphere: anterior-inferior, anterior-superior, posterior, tubular inferior, and tubular superior (Fig. 1). Each of these subunits includes two to five hypothalamic nuclei. Although the segmentation tool developed by Billot et al. (2020) was originally applied on raw, unprocessed T1-weighted images, we performed the segmentation on both raw and standardized images and compared the results. Visual inspection revealed that the segmentations from standardized images provided a lower failure rate and had clearer delineation of hypothalamic subunits, thereby improving the performance of the segmentation and facilitating the quality control process. As such, the analyses were performed using the volumes extracted from segmentations performed in standard space, after confirming the reliability of stereotactic volume calculations in relation to native space in a subset of the participants (see Supplementary Materials). All registrations were visually inspected to ensure accuracy.

Using FSLeyes, a tool from the FMRIB Software Library (Jenkinson et al., 2012), all individual segmentations (n = 245) were visually inspected for quality control based on a protocol described by Bocchetta et al. (2015). Segmentations with irregular shapes (n = 56), such as holes and fragmented subunits, were classified as low quality and excluded from further analyses (Supplementary Fig. S1). Additionally, we created a segmentation quality variable that we included as a covariate in our statistical models to further assess the robustness of our results. Segmentations displaying minor defects, such as the omission of a few voxels lining the third ventricle, were classified as moderate quality (n = 64). The remaining segmentations were classified as high quality (n = 125). Details regarding the quality assessment for each subunit are provided in Supplementary Table S1. In a subsequent step, the 64 moderate-quality segmentations were manually corrected using MNI Display, a software tool for visualization and manipulation of three-dimensional objects, integrated into the MNI MINC toolkit (http://www.bic.mni.mcgill.ca/ServicesSoftware/MINC). Analyses were then repeated using these corrected segmentations.

### 2.7 Statistical analyses

Participants’ demographic data, cardiometabolic variables, and changes in gastrointestinal appetite-regulating hormones following bariatric surgery were assessed using chi-squared tests for categorical variables and linear mixed-effects models for continuous variables. For the control group, demographic data and changes in hypothalamic volume between the first and the second pre-surgery visits were assessed using paired *t*-test. Linear mixed-effects models (Model I) were performed to examine hypothalamic volume changes following bariatric surgery. Participants were identified as categorical random variables, and age, sex, initial BMI, surgery type, and session were added as fixed effects. Post hoc tests were conducted for hypothalamic subregions that showed a significant main effect of time, in order to compare each post-surgery visit to the baseline measurement.

**Model I:** Hypothalamic volume ~ session + Age + Sex + BMI_baseline_ + Surgery type + (1| Participant)

Linear mixed-effects models (Model II) were used to examine the associations between cardiometabolic variables (total weight loss, percentage of fat mass, SBP, HOMA-IR, plasmatic levels of triglycerides, and HDL-C) and total left and right hypothalamus volume and their subunits. Participants were added to the model as categorical random variables, and age, sex, initial BMI, and surgery type as fixed effects.

**Model II:** Hypothalamic volume ~ cardiometabolic variables + Age + Sex + BMI_baseline_ + Surgery type + (1| Participant)

Linear mixed-effects models (Model III) were used to examine the associations between gastrointestinal appetite-regulating hormones (fasting total ghrelin and acylated ghrelin, as well as postprandial GLP-1 and PYY) and total left or right hypothalamus volume and their subunits. Participants were added to the model as categorical random variables, and age, sex, initial BMI, and surgery type as fixed effects.

**Model III:** Hypothalamic volume ~ gastrointestinal appetite-regulating hormones + Age + Sex + BMI_baseline_ + Surgery type + (1| Participant)

In Models II and III, the variables referred to as “cardiometabolic variables” or “gastrointestinal appetite-regulating hormones” reflect longitudinal measurements entered as absolute values collected at each time point (baseline, 4, 12, and 24 months), rather than baseline values or change scores. Due to the limited sample size and potential multicollinearity, each cardiometabolic or hormonal variable was examined in a separate model. An unstructured covariance matrix was used in all linear mixed-effects models to account for potential collinearity among repeated measures.

Two additional analyses were conducted to determine robustness of the segmentation tool in this population. First, analyses were carried out to assess differences between segmentation quality types. T-tests between high-quality and moderate-quality segmentation groups were used for normally distributed variables with equal variance, whereas Welch’s tests were used for unequal variance. Variables not following a normal distribution were first transformed with a log or a BoxCox transformation. If a normal distribution could not be achieved after transformations, a non-parametric test (Wilcoxon test) was used. Second, linear mixed-effects models (Model IV) were used to examine whether segmentation quality was associated with longitudinal changes in hypothalamic volume following bariatric surgery. Participants were identified as categorical random variables, and session, age, sex, initial BMI, surgery type, and segmentation quality were entered as fixed effects.

**Model IV:** Hypothalamic volume ~ segmentation quality + session + Age + Sex + BMI_baseline_ + Surgery type + (1| Participant)

In a second step, moderate-quality segmentations were manually corrected. Changes in hypothalamic volume were then re-assessed using the same linear mixed-effects models as initially applied (Model I).

Further analyses were performed. Total grey matter volume (GMV) and intracranial volume (ICV) were each independently included as fixed effects in separate iterations of Model I to control for total GMV and ICV. Total GMV was also added as a fixed effect in Models II and III to control for potential global GMV changes over time. Moreover, analyses were conducted to assess the effect of surgery type on fixed effects by adding an interaction term between surgery type and session in Model I, and between surgery type and gastrointestinal appetite-regulating hormones in Model III.

All results were corrected for multiple comparisons with the false discovery rate (FDR) approach using the Benjamini–Hochberg procedure (see Supplementary Materials). Statistical analyses were performed using JMP Pro version 16.0 (SAS Institute Inc., Cary, NC, USA) and graphs were produced using R version 4.4.2 (R Development Core Team, 2024).

## 3 Results

### 3.1 Clinical characteristics of the participants

Table 1 shows the characteristics of the participants. After quality control of the hypothalamus segmentations, a total of 73 participants (mean age 44.5 ± 9.1 years, mean BMI 43.5 ± 4.1 kg/m^2^) were included at baseline (Supplementary Fig. S1). The study population consisted mainly of females (71%). SG was the most frequently performed procedure. As expected, participants had significant weight loss following bariatric surgery (p < 0.0001). They also showed improvements in blood pressure (systolic and diastolic, both p < 0.0001), improvements in glucose homeostasis markers (fasting glycemia, fasting insulin, HOMA-IR index, and HbA1c, all p < 0.0001) and lipid profile (total cholesterol, p = 0.009; HDL-C, p < 0.0001; and triglycerides, p < 0.0001). Total and acylated fasting ghrelin levels were significantly decreased post-surgery (both p < 0.0001), while postprandial GLP-1 and PYY levels were significantly increased (p = 0.029 and p < 0.0001, respectively).

**Table 1.** Characteristics of participants.

	Baseline	4 months	12 months	24 months	p-value
N	73	60	34	22	—
Age (years)	44.5 ± 9.1	—	—	—	—
Sex (F: M)	52: 21	43: 17	27: 7	15: 7	0.777^a^
Type of surgery SG RYGB BPD-DS	47 10 16	42 9 9	20 8 6	13 5 4	—
Diabetes n (%)	19 (26)	17 (28)	10 (29)	5 (23)	0.942^a^
Weight (kg)	121.1 ± 14.6	98.1 ± 12.0	80.8 ± 12.4	81.9 ± 16.5	<0.0001^b^
BMI (kg/m^2^)	43.5 ± 4.1	34.2 ± 3.7	29.6 ± 4.5	29.3 ± 4.7	<0.0001^b^
Waist circumference (cm)	129.8 ± 10.2	111.4 ± 10.3	100.4 ± 11.1	99.2 ± 11.9	<0.0001^b^
Percentage of body fat mass (%)	47.0 ± 6.3	42.6 ± 7.7	36.8 ± 8.7	37.3 ± 8.4	<0.0001^b^
Total weight loss (%)	—	21.6 ± 4.0	32.8 ± 7.7	33.7 ± 7.9	<0.0001^b^
Excess weight loss (%)	—	46.7 ± 9.9	70.0 ± 17.1	71.9 ± 16.9	<0.0001^b^
SBP (mm Hg)	136 ± 16	123 ± 13	119 ± 12	119 ± 11	<0.0001^b^
DBP (mm Hg)	81 ± 11	74 ± 10	74 ± 11	71 ± 9	<0.0001^b^
Fasting glycemia^§^ (mmol/L)	6.3 ± 1.7	5.2 ± 1.0	4.8 ± 0.8	4.9 ± 0.8	<0.0001^b^
Fasting insulin^§^ (mU/L)	175.1 ± 95.7	69.0 ± 40.5	50.9 ± 34.5	41.4 ± 21.3	<0.0001^b^
HbA1c (%)^§^	5.77 ± 0.93	5.23 ± 0.50	5.14 ± 0.50	5.10 ± 0.15	<0.0001^b^
HOMA-IR index^§^	8.4 ± 5.5	2.8 ± 2.1	1.9 ± 1.6	1.6 ± 0.9	<0.0001^b^
Total cholesterol (mmol/L)	4.6 ± 0.9	4.0 ± 1.1	4.2 ± 0.8	4.4 ± 0.8	0.009^b^
LDL-cholesterol (mmol/L)	2.6 ± 0.8	2.3 ± 1.0	2.3 ± 0.8	2.2 ± 0.7	0.217^b^
HDL-cholesterol (mmol/L)	1.2 ± 0.3	1.2 ± 0.3	1.4 ± 0.3	1.6 ± 0.4	<0.0001^b^
Triglycerides (mmol/L)	1.7 ± 0.8	1.4 ± 1.2	1.1 ± 0.4	1.1 ± 0.4	<0.0001^b^
Fasting ghrelin (pg/ml) Total^§^ Acylated^§^	309 ± 215 61 ± 164	209 ± 116 55 ± 166	200 ± 127 31 ± 22	210 ± 153 28 ± 14	<0.0001^b^ <0.0001^b^
Postprandial GLP-1^§^ (pmol/L)	84 ± 63	114 ± 73	96 ± 60	105 ± 76	0.029^b^
Postprandial PYY (pmol/L)	31 ± 3	61 ± 5	66 ± 7	62 ± 9	<0.0001^b^

Values are presented as mean ± standard deviation or n (%). F: female, M: male, SG: sleeve gastrectomy, RYGB: Roux-en-Y gastric bypass, BPD-DS: biliopancreatic derivation with duodenal switch, BMI: body mass index, SBP: systolic blood pressure, DBP: diastolic blood pressure, GLP-1: glucagon-like peptide 1, PYY: peptide YY. ^a^Chi-squared test, ^b^mixed-effects models comparing baseline, 4, 12, and 24 months post-surgery sessions, ^§^log transformed.

No significant differences in age, sex, and surgery type were observed between the high-quality segmentation group and the moderate-quality segmentation group for all sessions (Supplementary Table S2). However, participants in the moderate-quality segmentation group had a higher BMI at baseline and a lower total weight loss at 12 months post-surgery compared with participants in the high-quality segmentation group (p = 0.0307 and p = 0.0226, respectively; Supplementary Table S2). A higher percentage of participants with type 2 diabetes was observed in the high-quality segmentation group than in the moderate-quality group at 4 months post-surgery (p = 0.0027, Supplementary Table S2).

### 3.2 Hypothalamic volume changes following bariatric surgery

**Fig. 2.**
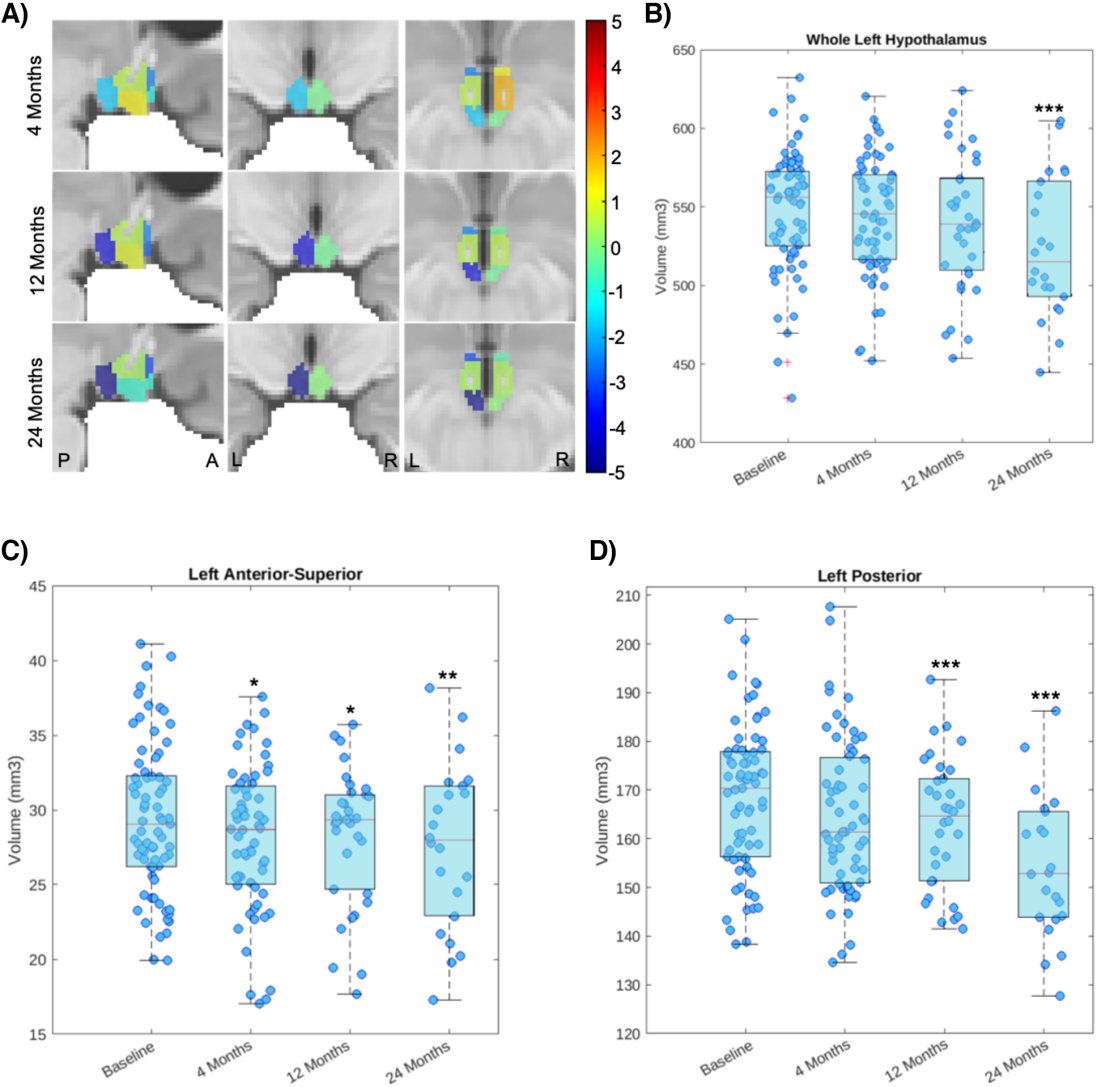
Changes in hypothalamus subunits 4, 12, and 24 months following bariatric surgery compared with baseline. Panel (A) shows the T-values from the mixed-effects models, contrasting the follow-up regional volumes against their baseline values. Warmer colors (e.g., orange and red) indicate an increase after surgery, while colder colors (e.g., blue and purple) indicate a decrease after surgery. “L” denotes left and “R” denotes right. “P” denotes posterior and “A” denotes anterior. Significant changes in volumes are observed in (B) the whole left hypothalamus, (C) the left anterior-superior subunit, and (D) the left posterior subunit. *p < 0.05, **p < 0.01, ***p < 0.001 versus baseline after FDR correction.

Figure 2A shows the T-values from the mixed-effects models (Model I), contrasting the follow-up regional volumes against their baseline values. Detailed results of the linear mixed-effects model for all regions are presented in Supplementary Table S3, and corresponding visual representations of longitudinal changes are shown in Supplementary Figures S2 and S3. A significant decrease in the whole volume of the left hypothalamus was observed 24 months post-surgery compared with baseline (p = 0.0007; Fig. 2B; Table 2). More specifically, a local decrease in volume was observed in the left anterior-superior subunit 4 months post-surgery compared with baseline (p < 0.05 after FDR correction; Fig. 2C; Table 2). This decrease in volume was maintained at 12 and 24 months post-surgery (p < 0.05 and p < 0.01 after FDR correction; Fig. 2C; Table 2). Additionally, a significant decrease in the volume of the left posterior subunit was also observed 12 and 24 months post-surgery compared with baseline (both p < 0.001 after FDR correction, respectively; Fig. 2D; Table 2). Supplementary Figure S4 shows the individual trajectories of participants for hypothalamic regions exhibiting significant volumetric changes. No significant changes in volume were found in the right hypothalamus and its subunits following bariatric surgery.

**Table 2.** Changes in hypothalamus subunits after bariatric surgery compared with baseline for hypothalamic subregions showing a significant main effect of time.

	Whole left			Left anterior-superior			Left posterior		
	β	S.E.	p-value	β	S.E.	p-value	β	S.E.	p-value
Intercept	540.06	52.77	**< 0.0001**	15.912	6.42	**0.0156**	137.66	19.40	**< 0.0001**
Age	-0.21	0.51	0.6858	0.07	0.06	0.2553	0.10	0.19	0.5885
Sex (male)	-3.70	5.04	0.4653	-0.68	0.63	0.2839	-3.13	1.87	0.0990
Baseline BMI	-0.15	1.16	0.8958	0.15	0.14	0.2886	0.23	0.42	0.5848
RYGB	-8.61	6.61	0.1970	-1.60	0.78	0.0452*	-4.94	2.33	0.0382*
BPD-DS	-2.10	5.77	0.7169	0.41	0.72	0.5724	-0.17	2.12	0.9365
4 months post-operatively	-0.63	1.63	0.7014	-0.48	0.19	**0.0146**	-1.23	0.70	0.0820
12 months post-operatively	-3.90	2.13	0.0724	-0.74	0.30	**0.0179**	-3.04	0.73	**0.0001**
24 months post-op	-8.22	2.19	**0.0007**	-1.04	0.33	**0.0039**	-6.28	1.24	**< 0.0001**

β values from the mixed-effects models for hypothalamic subunits showing a significant effect of time are presented (see Supplementary Table S2). Results that remained significant after FDR correction are shown in bold. BMI: body mass index, RYGB: Roux-en-Y gastric bypass, BPD-DS: biliopancreatic diversion with duodenal switch.

Furthermore, similar results were observed when total GMV was included as a covariate in Model I (Supplementary Table S4), as well as when ICV was added as a covariate (Supplementary Table S5). No significant interaction between surgery type and session was found across any hypothalamic subunit (Supplementary Table S6). No significant differences in hypothalamic volumes were found between the high- and moderate-quality segmentation groups after FDR correction (Supplementary Table S7). Adding the segmentation quality variable as a fixed effect (Model IV) did not affect the results regarding volume changes in the left and right hypothalamus or their subunits (Supplementary Table S8). Similar results were also obtained after manual correction of the moderate-quality segmentations, with significant decreases in volume observed for the left posterior subunit at both 12 and 24 months post-surgery (Supplementary Tables S9 and S10). However, unlike the uncorrected segmentations, no significant volume changes were detected in the total volume of the left hypothalamus and in the left anterior-superior subunit after FDR correction (Supplementary Table S9).

Characteristics of the control participants who completed two pre-surgery visits are given in Supplementary Table S11. Following quality control of the hypothalamus segmentations, 17 participants were included in the analysis. As expected, no significant changes in weight or cardiometabolic parameters were observed between pre-surgery sessions, except for SBP (p = 0.0098). No significant differences in hypothalamic volumes were observed in the control group between the two pre-surgery sessions after FDR correction (Supplementary Table S12).

### 3.3 Associations between changes in hypothalamic volumes and cardiometabolic variables following bariatric surgery

We used a mixed-effects model (Model II) to explore the associations between hypothalamic volumes and cardiometabolic variables (Table 3). Significant associations were found between the volume of the whole left hypothalamus and both the percentage of total weight loss (β = -39.88, p = 0.0008) and the percentage of fat mass (β = 1.06, p = 0.0021). Specifically, lower hypothalamic volume was associated with greater total weight loss and a lower percentage of fat mass. Associations between the percentage of total weight loss and specific subunits were also observed, including the left anterior-superior (β = -5.47, p = 0.0002) and left posterior subunits (β = -20.08, p < 0.0001). Conversely, no significant association was observed between changes in the volume of the right hypothalamus and weight loss.

**Table 3.** Associations between hypothalamic volumes and metabolic parameters following bariatric surgery.

	Left								
	Whole left			Anterior-inferior			Anterior-superior		
	β	S.E.	p-value	β	S.E.	p-value	β	S.E.	p-value
% Total weight loss	**-39.88**	**11.22**	**0.0008**	-3.83	1.63	0.0223*	**-5.47**	**1.38**	**0.0002**
% Fat mass	**1.06**	**0.33**	**0.0021**	0.10	0.05	0.0405*	**0.11**	**0.04**	**0.0082**
HDL-C	-18.37	7.36	0.0153*	-1.98	0.99	0.0488*	-2.61	0.95	0.0077*
Triglycerides	1.01	3.14	0.7484	0.41	0.47	0.3881	0.69	0.44	0.1204
Systolic blood pressure	0.24	0.13	0.0543	0.01	0.02	0.482	0.04	0.02	0.0147*
HOMA-IR	0.29	0.45	0.5118	0.05	0.05	0.3782	0.08	0.06	0.1439

	Posterior			Tubular inferior			Tubular superior		
	β	S.E.	p-value	β	S.E.	p-value	β	S.E.	p-value
% Total weight loss	**-20.08**	**4.18**	**< 0.0001**	-1.07	4.75	0.8234	-0.73	4.51	0.8721
% Fat mass	**0.51**	**0.12**	**0.0001**	0.06	0.14	0.654	0.02	0.14	0.8893
HDL-C	-5.06	3.29	0.1270	5.27	3.08	0.1027	-4.47	2.69	0.1037
Triglycerides	1.62	1.33	0.2257	-0.35	1.57	0.828	-0.62	1.27	0.6284
Systolic blood pressure	**0.16**	**0.05**	**0.0022**	-0.05	0.05	0.3335	0.07	0.05	0.1872
HOMA-IR	0.19	0.13	0.1712	-0.14	0.20	0.4988	-0.02	0.17	0.9145

	Right								
	Whole right			Anterior-inferior			Anterior-superior		
	β	S.E.	p-value	β	S.E.	p-value	β	S.E.	p-value
% Total weight loss	-1.98	11.33	0.8621	-2.75	1.75	0.1207	0.12	1.84	0.9487
% Fat mass	0.16	0.30	0.6046	0.01	0.05	0.8037	0.01	0.05	0.8358
HDL-C	-5.75	8.05	0.4772	-1.33	1.18	0.2640	-1.04	1.22	0.3926
Triglycerides	-1.12	3.00	0.7094	0.66	0.48	0.1768	0.03	0.48	0.9559
Systolic blood pressure	-0.002	0.13	0.9882	0.03	0.02	0.1341	-0.005	0.02	0.804
HOMA-IR	-0.52	0.44	0.2405	-0.06	0.06	0.3415	-0.02	0.06	0.785

	Posterior			Tubular inferior			Tubular superior		
	β	S.E.	p-value	β	S.E.	p-value	β	S.E.	p-value
% Total weight loss	-0.15	4.22	0.9719	-2.33	5.14	0.6522	5.86	5.62	0.3008
% Fat mass	0.06	0.12	0.6268	0.12	0.15	0.4207	-0.02	0.16	0.8796
HDL-C	-0.89	3.26	0.7870	-0.20	3.69	0.9579	-5.65	3.70	0.1295
Triglycerides	-0.50	1.38	0.7177	-0.36	1.43	0.8028	-0.64	1.45	0.6582
Systolic blood pressure	-0.01	0.05	0.7708	-0.05	0.05	0.4009	-0.007	0.06	0.9015
HOMA-IR	-0.08	0.17	0.621	-0.19	0.17	0.2702	-0.04	0.20	0.8542

β values from the mixed-effects models for the whole hypothalamus and its subunits. Results that remained significant after FDR correction, applied independently for each variable, across all subunits and across all time points (p < 0.05) are shown in bold. Results not significant following FDR correction are indicated with an asterisk (*). S.E.: standard error, HOMA-IR: homeostasis model assessment for insulin resistance, HDL-C: high-density lipoprotein cholesterol.

No significant associations were found between hypothalamic volumes and other lipid profile markers, including HDL-C, triglycerides, LDL-C, and total cholesterol (Table 3, results for LDL-C and total cholesterol not shown). A lower volume in the left posterior subunit was significantly associated with lower SBP levels (β = 0.16, p = 0.0022; Table 3). No significant associations were found between hypothalamic volumes and indicators of glucose homeostasis, namely HOMA-IR, fasting glycemia, and fasting insulin (Table 3, results for glycemia and insulin not shown). Including total GMV in Model II did not change the observed associations between hypothalamic volumes and cardiometabolic variables (Supplementary Tables S13–S18).

### 3.4 Associations between hypothalamic volumes and gastrointestinal appetitive hormone levels following bariatric surgery

Lower volumes in the left anterior-superior and in the left posterior subunits were associated with higher postprandial PYY levels (β = -0.03, p = 0.0034 and β = -0.10, p = 0.0001, respectively; Table 4). The volume of the right anterior-inferior subunit showed positive associations with total and acylated ghrelin levels; however, these associations did not remain significant after FDR correction (Table 4). Lower volumes in the left posterior and right anterior-inferior subunits were also negatively associated with higher postprandial GLP-1 levels (β = -0.04, p = 0.0119 and β = -0.01, p = 0.0076, respectively, after FDR correction; Table 4). Consistent with other models, all associations in Model III followed the same trend after the inclusion of total GMV (Supplementary Tables S19–S22), and no significant interaction between surgery type and gastrointestinal hormones was found across all subunits (Supplementary Tables S23–S26).

**Table 4.** Associations between hypothalamic volumes and variations in gastrointestinal appetite-regulating hormone levels following bariatric surgery.

	Left								
	Whole left			Anterior-inferior			Anterior-superior		
	β	S.E.	p-value	β	S.E.	p-value	β	S.E.	p-value
Ghrelin, total	5.28	4.41	0.2337	0.39	0.56	0.4907	0.33	0.55	0.5516
Ghrelin, acylated	3.97	4.75	0.4043	0.12	0.56	0.8303	0.46	0.61	0.4530
GLP-1	-0.04	0.03	0.2490	0.005	0.005	0.3266	-0.002	0.005	0.6376
PYY	-0.11	0.07	0.0918	0.007	0.009	0.4346	**-0.03**	**0.009**	**0.0034**

	Posterior			Tubular inferior			Tubular superior		
	β	S.E.	p-value	β	S.E.	p-value	β	S.E.	p-value
Ghrelin, total	1.50	1.79	0.4027	1.45	1.92	0.4545	-1.04	1.77	0.5567
Ghrelin, acylated	-0.57	1.92	0.7654	3.91	2.00	0.0538	-0.34	1.76	0.8488
GLP-1	**-0.04**	**0.01**	**0.0119**	-0.005	0.01	0.7087	-0.01	0.01	0.3292
PYY	**-0.10**	**0.02**	**0.0001**	0.01	0.03	0.6260	-0.03	0.03	0.2780

	Right								
	Whole right			Anterior-inferior			Anterior-superior		
	β	S.E.	p-value	β	S.E.	p-value	β	S.E.	p-value
Ghrelin, total	1.44	4.5	0.7500	1.71	0.61	0.0055*	-0.02	0.60	0.9796
Ghrelin, acylated	1.20	4.84	0.8051	1.39	0.60	0.0234*	0.31	0.61	0.6056
GLP-1	-0.09	0.03	0.0057*	**-0.01**	**0.005**	**0.0076**	-0.001	0.005	0.7115
PYY	-0.07	0.07	0.2819	-0.01	0.01	0.1505	-0.001	0.01	0.8899

	Posterior			Tubular inferior			Tubular superior		
	β	S.E.	p-value	β	S.E.	p-value	β	S.E.	p-value
Ghrelin, total	-0.96	1.66	0.5665	2.22	1.88	0.2406	-1.11	1.80	0.5397
Ghrelin, acylated	-1.99	1.93	0.3038	2.15	1.99	0.2827	-0.62	1.82	0.7363
GLP-1	-0.02	0.01	0.0856	-0.03	0.01	0.0223*	-0.02	0.01	0.1376
PYY	-0.03	0.03	0.3081	-0.02	0.03	0.4311	-0.02	0.03	0.4003

β values from the mixed-effects models for the whole hypothalamus and its subunits. Results that remained significant after FDR correction, applied independently for each variable, across all subunits, and across all time points (p < 0.05) are shown in bold. Results not significant following FDR correction are indicated with an asterisk (*). Ghrelin (total and acylated) was measured after a 12-hour fast, while GLP-1 and PYY were measured in postprandial. S.E.: standard error, GLP-1: glucagon-like peptide 1, PYY: peptide YY.

## 4 Discussion

In this study, a tool based on a deep convolutional neural network was used to examine hypothalamic volume changes following significant weight loss induced by bariatric surgery. Our findings revealed reduced volumes of the whole left hypothalamus, specifically in the left anterior-superior and left posterior subunits. These hypothalamic morphological changes post-surgery were associated with the percentage of total weight loss, as well as with metabolic improvements, namely SBP. We also examined the associations between hypothalamic volumes and gastrointestinal appetite-regulating hormone levels to explore potential mechanisms underlying hypothalamic alterations. We found that lower volumes in the left anterior-superior and left posterior subunits were associated with higher postprandial PYY levels.

To our knowledge, only one recent study has assessed hypothalamic volume following bariatric surgery using a deep convolutional neural network method (Pané et al., 2024). They found reduced volume in the whole left hypothalamus and its subunits 1 year post-surgery, with no significant changes in the whole right hypothalamus, which aligns with our findings. However, they reported more widespread changes in hypothalamic volumes compared with our study. Differences in the population studied and the types of bariatric surgeries performed could potentially explain the variations in results. Furthermore, our relatively small sample size, particularly at the 24-month follow-up due to participant attrition, may have limited our statistical power to detect significant changes in hypothalamic subunit volumes.

The posterior subunit notably encompasses the lateral nuclei. The orexin neurons within these nuclei are well known for their role in food intake (Toshinai et al., 2003). Studies have reported bilateral increases in the posterior subunits in obesity (Alzaid et al., 2024; Brown et al., 2023). However, our results show that only the volume of the left posterior subunit is reduced following bariatric surgery. This decrease in volume 12 and 24 months post-surgery persists when controlling for segmentation quality, GMV, or total ICV in the statistical models, as well as after manual correction of the moderate-quality segmentations, thereby confirming the robustness of this finding. Moreover, similar results were reported in another study assessing hypothalamic changes following bariatric surgery (Pané et al., 2024). This suggests that alterations observed in obesity are reversible only for the left side. Lateralization of the hypothalamus has also been reported in other volumetric studies using the segmentation tool developed by Billot et al. (2020) (Pané et al., 2024; Ruzok et al., 2022) as well as in a study using semi-automated segmentation methods (Thomas et al., 2019). Studies using various MRI techniques have also reported hypothalamic inflammation predominantly in the left hemisphere (Kreutzer et al., 2017; Schur et al., 2015; Thomas et al., 2019). Moreover, pathways involving the hypothalamus and other brain regions implicated in homeostatic feeding are suggested to be lateralized to the left side (Castro et al., 2015; Kiss et al., 2020). Although numerous studies in the literature suggest that neurobiological differences may underlie this asymmetry, non-biological factors could also have influenced our findings. The quality assessment metrics obtained in our study indicate that technical or segmentation-related issues affected the right hemisphere more frequently than the left.

Additionally, we found a reduced volume in the left anterior-superior subunit at 4, 12, and 24 months following bariatric surgery. Similar results were reported by Pané et al. (2024), as they also observed reduced volume in this subunit 12 months post-surgery. The left anterior-superior subunit includes parts of the paraventricular nucleus, which plays a role in food intake through second order neurons that receive projections from orexinergic neurons expressing neuropeptide Y (NPY)/ agouti-related peptide (AgRP) of the arcuate nucleus (Li et al., 2023; Roger et al., 2021). Interestingly, evidence shows a higher total volume in the anterior-superior subunits (including both left and right parts) in participants living with obesity as compared with lean controls (Alzaid et al., 2024). However, another study found no difference when examining the left and right sides independently (Brown et al., 2023). These discordant results may be explained by the small size of this subunit. In addition, while reduced volumes remain when controlling for the segmentation quality, no more significant changes are observed after manual correction of moderate-quality segmentations. This suggests that this small subunit may be more prone to temporal variability. Indeed, given the small size of this subunit, it is plausible that even minimal adjustments had a proportionally larger impact on volume estimates. Moreover, segmentation of the anterior-superior and anterior-inferior subunits is reported to have lower accuracy due to the challenging boundary between them (Billot et al., 2020). Therefore, those results need to be interpreted with caution.

Few studies have reported increased hypothalamic volume of the tubular inferior subunit among participants with obesity compared with those with normal BMI (Alzaid et al., 2024; Pané et al., 2024). This subunit includes, among others, the arcuate nucleus and the ventromedial nucleus, both known for their roles in homeostatic feeding. Our results showed no significant volume differences in this subunit following bariatric surgery, contrasting with another study that found decreased volume in individuals living with obesity but without type 2 diabetes, with no changes observed in those with type 2 diabetes (Pané et al., 2024). These differences suggest that some clinical characteristics and inter-individual variability may influence the reversibility of hypothalamic alteration in the arcuate nucleus in the short term, which could explain the lack of volume changes observed in our study. The tubular inferior subunit also includes four other nuclei, potentially explaining the absence of volume changes post-surgery in our study. The absence of recuperation of this structure could also provide insights into the mechanisms explaining frequent weight regain observed in the years following bariatric surgery. Therefore, it is important to address this gap in our current knowledge, and further studies should aim to determine whether these alterations can be reversed over a longer period.

Interestingly, structural brain changes following weight loss and cardiometabolic improvements may be region specific. In a previous study, we reported increased grey matter density, mainly in the cerebellum, occipital and temporal cortices, up to 24 months post-bariatric surgery (Legault et al., 2024; Michaud et al., 2020). In contrast, the present findings show a reduction in hypothalamic volume, suggesting a distinct regional response, potentially related to the resolution of gliosis or edema (Pané et al., 2024). This supports the idea that different brain regions may respond uniquely to metabolic changes, depending on their function, cellular composition, and degree of exposure to metabolic signals.

Several mechanisms have been proposed to explain hypothalamic alterations associated with obesity. Most evidence points toward hypothalamic inflammation and gliosis, which have been demonstrated in preclinical studies and confirmed in humans using neuroimaging studies (Sewaybricker et al., 2023). Neuroinflammation is known to lead to production of proteases and free radicals that can affect the integrity of multiple brain structures. For instance, proteases could disrupt the tight junctions of the blood–brain barrier, leading to buildup of extracellular fluid and subsequent increases in volume (Rosenberg & Yang, 2007). This is consistent with the increased mean diffusivity in the hypothalamus observed in obesity, which has been reported with concomitant increased volumes (Pané et al., 2024). Moreover, alterations in perineuronal nets, an extracellular matrix protecting synapses, have been observed in rodents on a high-fat, high-sugar diet (Reichelt et al., 2019). Without the protection of perineuronal nets, neurons are more exposed to free radicals, potentially leading to hypothalamic inflammation and increased volume. As bariatric surgery leads to decreased systemic inflammation (Askarpour et al., 2019) and reduced hypothalamic inflammation (Hankir et al., 2019; Pané et al., 2024), this reduction could explain the following decrease in hypothalamic volume post-surgery. However, our study was not designed to explore the pathways underlying these volume changes. Future studies are, therefore, needed to elucidate these intriguing mechanisms.

Many associations were found between changes in hypothalamic subunit volumes and greater weight loss following surgery, as well as improvements in cardiometabolic health, including a better blood pressure control. However, no significant association was observed between structural changes in the hypothalamus and glycemic control, as assessed with HOMA-IR. This lack of association may be due to several factors. As HOMA-IR primarily reflects peripheral insulin resistance, it may not capture central mechanisms relevant to hypothalamic structure. Moreover, hypothalamic changes may be more responsive to direct hormonal signaling, such as gastro-intestinal hormones, rather than metabolic indices such as HOMA-IR. Finally, only approximately a quarter of our cohort had impaired glucose tolerance, which may have limited the statistical power to detect such associations.

To our knowledge, our study is the first to explore the associations between hypothalamic volume changes and gastrointestinal appetite-regulating hormone levels after bariatric surgery.

We found that higher postprandial PYY levels were associated with greater reductions in the left anterior-superior (which encompasses the preoptic and paraventricular nuclei) and left posterior (which includes the lateral nucleus) subunits post-surgery. These findings suggest region-specific effects of PYY on hypothalamic structure. The primary receptor for PYY (3–36), Y2, is mostly co-localized with NPY neurons in the arcuate nucleus and is also expressed in the preoptic and dorsomedial nuclei (Broberger et al., 1997). Our findings align with a previous fMRI study showing increased activity in the posterior hypothalamus following PYY administration in humans (Batterham et al., 2007) and in an animal study showing PYY immunoreactivity in the paraventricular nucleus (Morimoto et al., 2008). These findings support the hypothesis that PYY may exert indirect effects on the lateral and paraventricular nuclei through its action on the arcuate nucleus, highlighting the need for further research to examine hypothalamic responses to metabolic signals.

We also found a negative association between GLP-1 levels and volumes of the left posterior and right anterior-inferior hypothalamic subunits. This direction of association is consistent with known physiological changes, as hypothalamic subunit volumes tend to decrease after bariatric surgery, while GLP-1 levels typically increase (Huang et al., 2023).

Several limitations need to be acknowledged. The tool we used did not allow for the segmentation of individual nuclei. Subunits include two to five nuclei, and some nuclei are divided into two different subunits, thus restricting the interpretation of the results. Our study did not include any peripheral or cerebral markers of inflammation, nor did it incorporate MRI sequences to assess brain inflammation, a potentially critical mechanism suggested to participate in the remodeling of the hypothalamus. Thus, we cannot assess the role of inflammation in hypothalamic volume changes. While this is the first study to examine long-term changes in hypothalamic structure and subregions following bariatric surgery using MRI scans up to 2 years post-surgery, the cohort completing the full 2-year follow-up was relatively small. In addition, given the small size of our sample, we were unable to assess differences in our primary outcome across the three types of surgery. Nonetheless, surgery type was included as a covariate in our statistical models to adjust for potential confounding, and was also tested as an interaction term with session to explore whether longitudinal changes varied by surgical procedure.

## 5 Conclusion

In conclusion, our study shows reductions in volumes of the left hypothalamus and some specific left subunits following bariatric surgery, suggesting a recovery of hypothalamic alterations associated with obesity. Volume reductions were observed in the left posterior subunit, housing the lateral nuclei. This hypothalamic nucleus plays a crucial role in regulating homeostatic food intake. Our findings suggest that changes in the hypothalamus in post-bariatric surgery may be involved in shifts that could influence food intake regulation and potentially impact weight loss outcomes.

## Supplementary Material

Supplementary Figures

Supplementary Tables

## Data Availability

The automated hypothalamus segmentation method is publicly available at: https://github.com/BBillot/hypothalamus_seg. Data will be made available on request.
