# Supplementary material for: Changes in hypothalamic subunits volume and their association with metabolic parameters and gastrointestinal appetite-regulating hormones following bariatric surgery
Source: Imaging Neurosci (Camb). 2025 Oct 31;3:IMAG.a.970. doi: 10.1162/IMAG.a.970 (PMC12580804; doi:10.1162/IMAG.a.970)
Supplement: Supplementary Figures [file IMAG.a.970_supp.pdf]

Changes in hypothalamic subunits volume and their association with metabolic parameters and gastrointestinal appetite-regulating hormones following bariatric surgery

Supplemental files

Amélie Lachance[1,2,3], Justine Daoust[1,2,3], Mélissa Pelletier[2], Alexandre Caron[2], André C. Carpentier[4], Laurent Biertho[5], Josefina Maranzano[6], André Tchernof[2,3], Mahsa Dadar[7], Andréanne Michaud[1,2,3]

Affiliations

1.Centre Nutrition, Santé et Société (NUTRISS), Institut sur la nutrition et les aliments fonctionnels (INAF), Université Laval, Québec, Qc, G1V 0A6, Canada;

2. Institut universitaire de cardiologie et de pneumologie de Québec - Université Laval, Québec, Qc, G1V 4G5, Canada;

3. École de nutrition, Faculté des sciences de l'agriculture et de l'alimentation, Université Laval, Québec, Qc, G1V 0A6, Canada;

4. Centre de recherche du centre hospitalier universitaire de Sherbrooke, Université de Sherbrooke, Sherbrooke, Qc, J1H 5N4, Canada;

5. Département de chirurgie générale, Institut universitaire de cardiologie et de pneumologie de Québec-Université Laval, Québec, Qc, G1V 4G5, Canada;

6. Département d'anatomie, Université du Québec à Trois-Rivières, Trois-Rivières, Qc, G8Z 4M3, Canada;

7. Douglas Research Centre, Université McGill, Montréal, Qc, H4H 1R3, Canada.

Corresponding author: Andréanne Michaud, R.D., Ph.D. Address: 2725, Chemin Ste-Foy, Québec (QC), Canada, G1V 4G5. E-mail: andreanne.michaud@criucpq.ulaval.ca

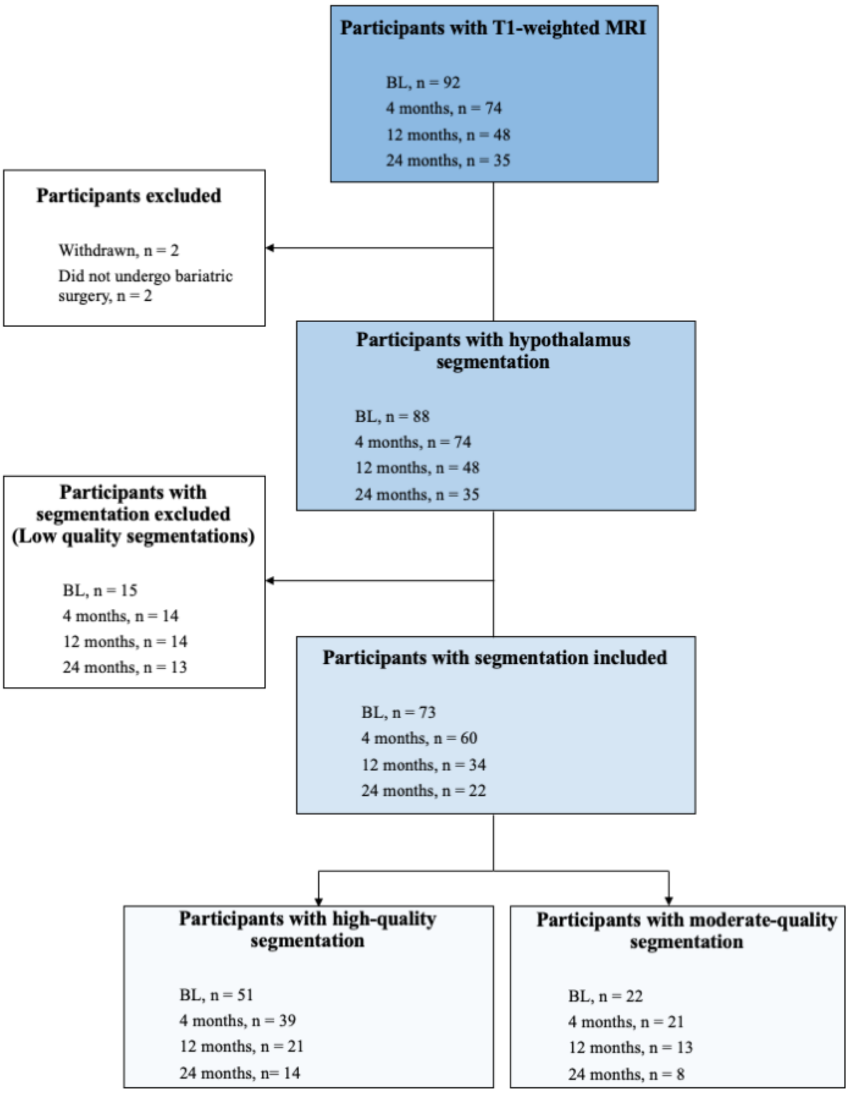

Figure S1. Flow-chart of participants included in the study.
BL: baseline.

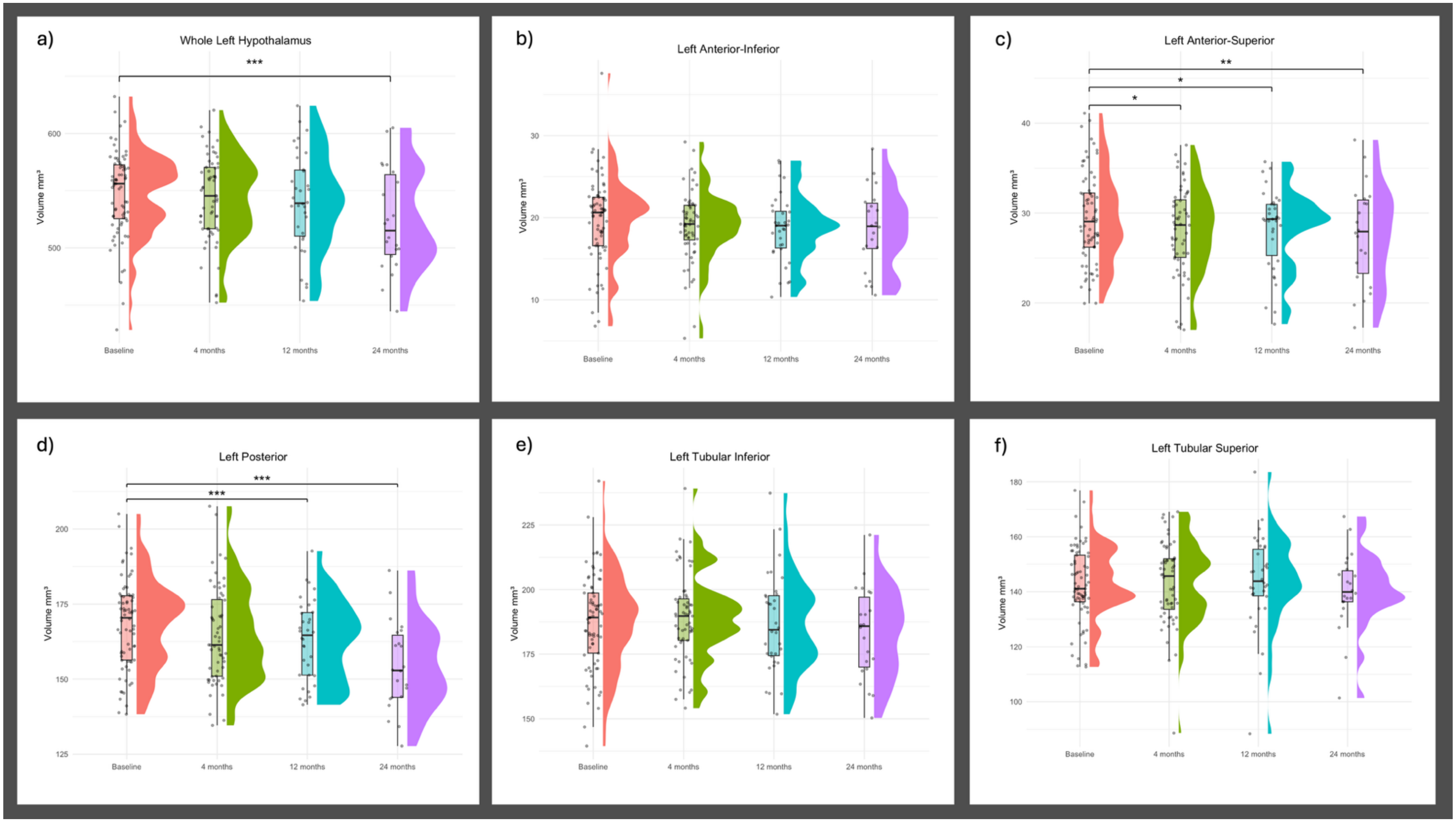

Figure S2. Distribution and longitudinal evolution of volumes for each left hypothalamic subunit and the whole left hypothalamus across all sessions

a) Whole left hypothalamus, b) Left Anterior-Inferior, c) Left Anterior-Superior, d) Left Posterior, e) Left Tubular Inferior, f) Left Tubular Superior. * $p < 0.05$, ** $p < 0.01$, *** $p < 0.001$ versus baseline *after FDR correction (p<0.05).*

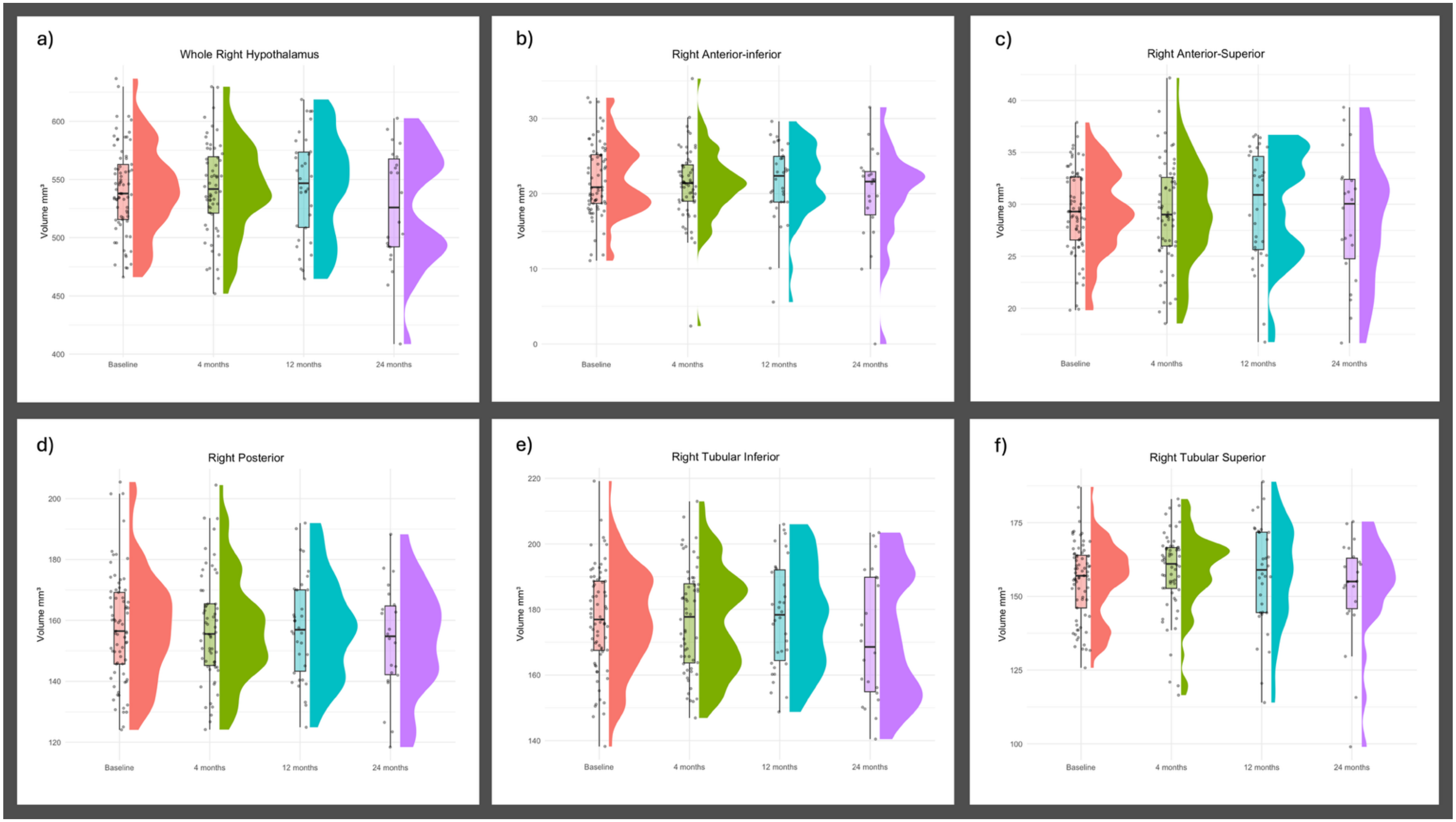

Figure S3. Distribution and longitudinal evolution of volumes for each right hypothalamic subunit and the whole right hypothalamus across all sessions

a) Whole right hypothalamus, b) Right Anterior-Inferior, c) Right Anterior-Superior, d) Right Posterior, e) Right Tubular Inferior, f) Right Tubular Superior. * $p < 0.05$, ** $p < 0.01$, *** $p < 0.001$ versus baseline *after FDR correction (p<0.05).*

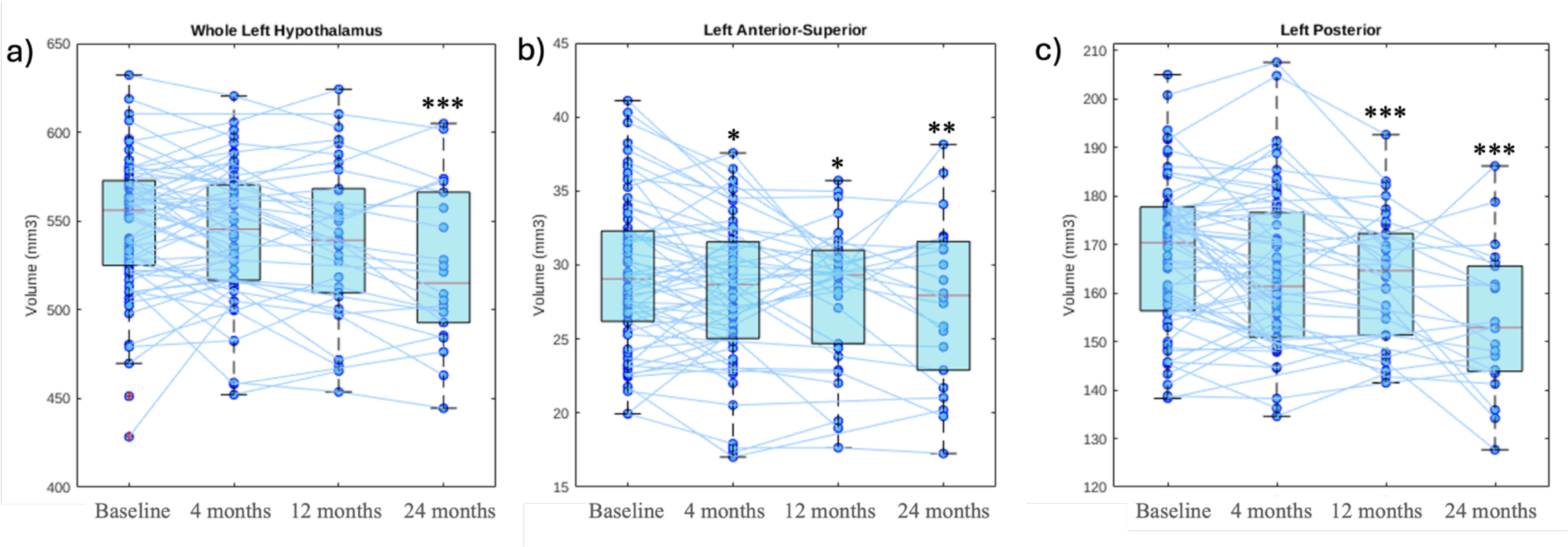

Figure S4. Within-subject volume trajectories at 4, 12 and 24 months following bariatric surgery compared to baseline, for hypothalamus subregions showing a significant main effect of time.
a) Whole left hypothalamus, b) Left Anterior-Superior, c) Left Posterior. * $p < 0.05$, ** $p < 0.01$, *** $p < 0.001$ versus baseline *after FDR correction ($p<0.05$).*
